# Straightforward and precise approach to replicate complex hierarchical structures from plant surfaces onto soft matter polymer

**DOI:** 10.1098/rsos.172132

**Published:** 2018-04-18

**Authors:** Charchit Kumar, Vincent Le Houérou, Thomas Speck, Holger F. Bohn

**Affiliations:** 1 Plant Biomechanics Group Freiburg, Botanic Garden, Faculty of Biology, University of Freiburg, Schänzlestraße 1, 79104 Freiburg, Germany; 2 Institut Charles Sadron (ICS), CNRS UPR022, Université de Strasbourg, 23 rue du Loess, BP 84047, 67034 Strasbourg Cedex 2, France; 3 Freiburg Centre for Interactive Materials and Bioinspired Technologies (FIT), Georges-Köhler-Allee 105, D-79110 Freiburg, Germany

**Keywords:** biological surfaces, replication, hierarchical microstructures, epoxy mould, soft matter, PDMS replica

## Abstract

The surfaces of plant leaves are rarely smooth and often possess a species-specific micro- and/or nano-structuring. These structures usually influence the surface functionality of the leaves such as wettability, optical properties, friction and adhesion in insect–plant interactions. This work presents a simple, convenient, inexpensive and precise two-step micro-replication technique to transfer surface microstructures of plant leaves onto highly transparent soft polymer material. Leaves of three different plants with variable size (0.5–100 µm), shape and complexity (hierarchical levels) of their surface microstructures were selected as model bio-templates. A thermoset epoxy resin was used at ambient conditions to produce negative moulds directly from fresh plant leaves. An alkaline chemical treatment was established to remove the entirety of the leaf material from the cured negative epoxy mould when necessary, i.e. for highly complex hierarchical structures. Obtained moulds were filled up afterwards with low viscosity silicone elastomer (PDMS) to obtain positive surface replicas. Comparative scanning electron microscopy investigations (original plant leaves and replicated polymeric surfaces) reveal the high precision and versatility of this replication technique. This technique has promising future application for the development of bioinspired functional surfaces. Additionally, the fabricated polymer replicas provide a model to systematically investigate the structural key points of surface functionalities.

## 1. Introduction

Surface functionalities are prominent when two counterparts come into contact and play an important role in the system's performance and efficiency [1–3]. This stands for most physical human-made as well as biological systems and two major parameters have to be taken into consideration: surface structuring and chemistry [4,5]. It is often crucial to fine-tune the frictional and adhesive properties of manufactured systems, when the surface-to-volume ratio gets tremendously increased in micro-contact applications (MEMS devices). For instance, a low adhesion for easy attachment and detachment of the devices is desired [6,7]. In this context, the surface micro- and nano-structuring is a possible solution to achieve such characteristics, besides altering the surface chemistry [7–10]. Surface structuring could also influence other surface properties of the technical systems such as optical effects, wetting, fluid flow, heat transfer, antifouling, etc. [11–14].

In nature, surfaces of plant leaves are often organized with a large variety of surface structuring; over a wide size range (from nano- to macro-scale), with distinct morphologies, and including several hierarchical levels [3,15,16]. Apart from surface chemistry, the specific and unique surface structuring gives rise to various remarkable and inspiring functionalities. For instance, self-cleaning behaviour of the sacred lotus (*Nelumbo nucifera*) leaves arise from the remarkable de-wetting ability due to the complex hierarchical structure composed of papillate epidermal cells covered with randomly oriented hydrophobic nano-scale wax crystals [17]. Anti-adhesive properties for insects attachments of the rubber tree (*Hevea brasiliensis*) leaves result from their fine micro-structuring, and the insect trapping in some carnivorous plants (e.g. *Nepenthes alata* Blanco) is caused by hierarchical structured architecture [18–20]. Within this context, biological surface structures may serve as role model to develop biomimetic and bioinspired surfaces and devices [21]. Furthermore, the production of precise polymer replicas of biological surfaces may help by allowing the investigation of structure influence on surface functionality, independently to surface chemistry [4,5].

The most commonly used technique to replicate structures from biological surfaces follows a two-step double casting process [5,22–32]. At first a negative mould is developed from a plant leaf (bio template); afterwards, this negative mould is used to transfer the structures onto the positive replica. In recent years, various approaches have been investigated to perform plant leaf replication (with different combinations of material for negative mould and positive replica). Williams *et al.* [22] introduced a technique with using polyvinyl siloxane (PVS) dental impression material as a negative mould and epoxy for the positive replica (hard material), and later this technique has been extensively investigated by other researchers [5,23,24]. In the PVS-epoxy approach, the fresh leaf was covered with PVS mixture and manually pressed down with a flat slide. The advantage of using PVS consists of its low adhesion to plant leaves which is useful to prevent artefacts. On the other hand, the quick polymerization of PVS (approximately in 2–5 min) constitutes a limitation for this technique as it could cause air to get trapped at the interface of mixture and leaf surface [23]. In another similar replication attempt, positive replicas were developed on PMMA (polymethyl methacrylate) from PDMS (polydimethylsiloxane) negative mould [25]. Here, plant leaves were exposed to high temperature during the development of negative mould. Shrinkage of the cell pattern can be seen on replicated surface, and final replicas were obtained on hard material (PMMA). Lee & Kwon [26] described an alternative approach to replicate the surface structure of bamboo leaf onto UV curable photopolymer, by using a hard negative mould of nickel. In this method, the substrate surface was first metallized by using gold sputtering and patterned by nickel electroforming [26]. For gold sputter coating, the substrate needs to be exposed under vacuum, which might induce cell shrinkage artefacts (surface distortion) [33,34]. Another replication approach has been described using PDMS to produce both the negative mould and the positive replica (PDMS–PDMS) [29,31]. The fresh leaf was filled up with PDMS mixture and cured at high temperature 80–90°C to develop a negative mould [29,30]. Again, the treatment of plant material with high temperatures is likely to facilitate shrinkage or collapse of surface structures resulting from evaporation of water from the cells. The strong adhesion (stiction) between PDMS mould and PDMS replica is the major issue for PDMS–PDMS replication, which could be overcome by creating an anti-stiction layer (organosilane monolayer deposition) on the PDMS mould [29,31,35]. In order to perform a silane deposition, the PDMS mould needs to be treated under plasma, a procedure that can cause surface damage or instability [36]. Furthermore, during silane vapour deposition, aggregations of silane molecules can induce surface roughness [37,38]. Finally, an anti-stiction surface treatment degrades with time and replication cycles. In general, an anti-stiction treated PDMS mould can be well used to develop three or four positive replicas [35]. However, the PDMS–PDMS approach is not well explored yet for developing replica from complex biological structured surfaces with typically undercut and overhanging patterns. One may note that the PDMS, a soft matter polymer, is an interesting candidate for positive replicas because it has been extensively studied and is a widely used material in many nano- and micro-technology applications: it has easy handling, low cost, wide commercial availability and non-toxicity [39–41]. Furthermore, it offers numerous fascinating characteristics such as: high optical transparency, a low surface energy (22 mJ m^−2^), excellent biocompatibility, chemical stability, high flexibility due to an extremely low elastic modulus (*E* ≃ 0.5–4 MPa, tunable with varying cross-linker density) [41–46].

In this paper, we present a new straightforward, inexpensive, robust and precise approach to replicate the microstructures directly from natural plant leaves in a hard and durable negative mould. It can be used as template for numerous positive soft matter replicas showing very precisely the original microstructure of the leaf surface. In this replication approach, epoxy resin (hard material) is used to develop negative mould and final positive replica is fabricated on the PDMS soft matter (hereinafter called the Epoxy–PDMS replication). A new alkaline chemical treatment is also established to wholly separate the leaf out from the negative mould in case of complex surface structures. In order to investigate the capabilities of this replication method, three different types of leaves were selected with different sizes (fine and coarse), distinct shapes and complexity (hierarchy) of their surface structures.

## 2. Material and methods

### 2.1. Plant materials

Three different model plant leaves were selected in this study: *H. brasiliensis* (Rubber tree), *Litchi chinensis* (Lychee) and *Ludisia discolor* (Jewel orchid). The leaves were chosen according to the different sizes (in the range of 0.5 µm to few 100 µm) and distinct morphology of their surface microstructures (figure 2). *L. discolor* (adaxial, i.e. upper leaf surface) represents coarse microstructures (circular cone-like shape structures with a height of about 50 µm and a diameter 50–100 µm). *H. brasiliensis* (adaxial, i.e. upper leaf surface) shows fine cuticular folds with both thickness and height of about 0.5–1 µm, and an intermediate spacing of 0.5–1.5 µm [47]. *L. chinensis* (abaxial, i.e. lower leaf surface) has a complex hierarchical surface structuring consisting of undercuts and overhanging substructures of approximately 0.5–1 µm in thickness. All plants from which leaf samples were taken are cultivated in the Botanic Garden of the University of Freiburg, Germany. In order to keep safe natural surface features and to avoid dehydration artefacts, plant leaves were freshly picked just before each replication process. Before replication, leaf surfaces were gently washed with distilled water to remove contaminations. Immediately after washing, the leaf surfaces were carefully dried with pressurized air.

### 2.2. Replication procedure

**Figure 1.**
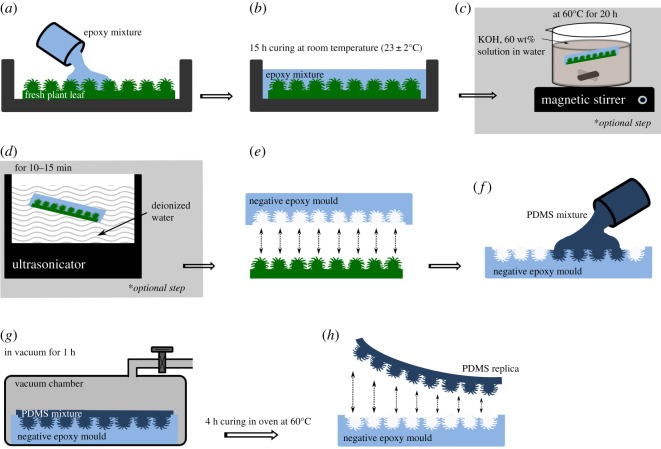
Schematic sketch of the two-step replication process. (*a*) Fresh plant leaf glued on a plastic Petri dish, filled up with epoxy resin. (*b*) Curing of epoxy mixture for 15 h to produce negative epoxy mould. (*c*)* Epoxy sample (which adhered with leaf surface) is kept for chemical treatment in potassium hydroxide solution on magnetic stirrer (at 60 ± 3°C for 20 h). (*d*)* Chemically treated sample washed in deionized water using an ultrasonicator. (*e*) Negative epoxy mould separated from the leaf surface. (*f*) Negative epoxy mould filled up with PDMS mixture. (*g*) Degassed in vacuum chamber to remove air trapped at the interface. (*h*) PDMS-positive replica peeled off from the epoxy mould. * Step *c* and *d* only necessary for complex hierarchical structured surface of *L. chinensis* leaves.

The replication technique proposed in this study follows a two-step process: at first, a hard epoxy negative mould was produced directly from plant leaf, and then patterns from the negative mould were replicated onto PDMS surfaces. A schematic representation of the replication procedure is shown in figure 1. First, small pieces of approximately 3.5 cm × 3.5 cm (larger or smaller area of sample can also be selected, the given sample area was chosen on purpose) were cut out from cleaned leaves and carefully attached onto a plastic Petri dish using double-side adhesive tape (tesa SE, Norderstedt, Germany). The two components epoxy resin (Epoxy Resin L & Hardener S, Toolcraft, Conrad Electronic SE, Hirschau, Germany) were uniformly mixed (mixing ratio of resin to hardener of 10 : 4.8) in a plastic cup for 3–5 min, using a glass rod. The mixture was then degassed in a vacuum chamber for 15 min to remove any dissolved and trapped air bubbles from the mixture. Afterwards, the epoxy resin was slowly and steadily (to avoid any bubbles) poured onto the leaf surface, so that mixture flows all over the Petri dish (figure 1*a*). The Petri dish filled with the epoxy resin is then kept for curing at room temperature (23 ± 2°C) for 15 h (figure 1*b*). Then the leaves were carefully peeled off from the cured epoxy negative moulds. During the peeling process, *H. brasiliensis* and *L. discolor* leaves were smoothly and wholly separated from cured negative epoxy moulds (figure 1*e*), whereas, in the case of *L. chinensis*, the leaf surface was strongly embedded in the cured epoxy mould and could not be peeled off undamaged. To separate *L. chinensis* leaf out from the cured negative epoxy mould, an alkaline chemical solution treatment was performed (figure 1*c*,*d*). A potassium hydroxide solution (KOH, ≥85%, p.a., Carl Roth GmbH & Co. KG, Karlsruhe, Germany) was prepared in distilled water at a concentration of 60 g/100 ml. The cured negative epoxy mould along with the *L. chinensis* leaf still attached was kept in a closed beaker with the KOH solution at 60 ± 3°C with a magnetic stirrer running at 450 ± 25 r.p.m. for 20 h (figure 1*c*). The sample was then removed from the solution and placed in an ultrasonicator (in deionized water) for 10–15 min (figure 1*d*). The leaf was carefully cut along the edges of the mould using a scalpel and then peeled off (figure 1*d*). Afterwards the negative mould was blown with pressurized air in order to dislodge any leftover particles and also to dry the mould.

**Figure 2.**
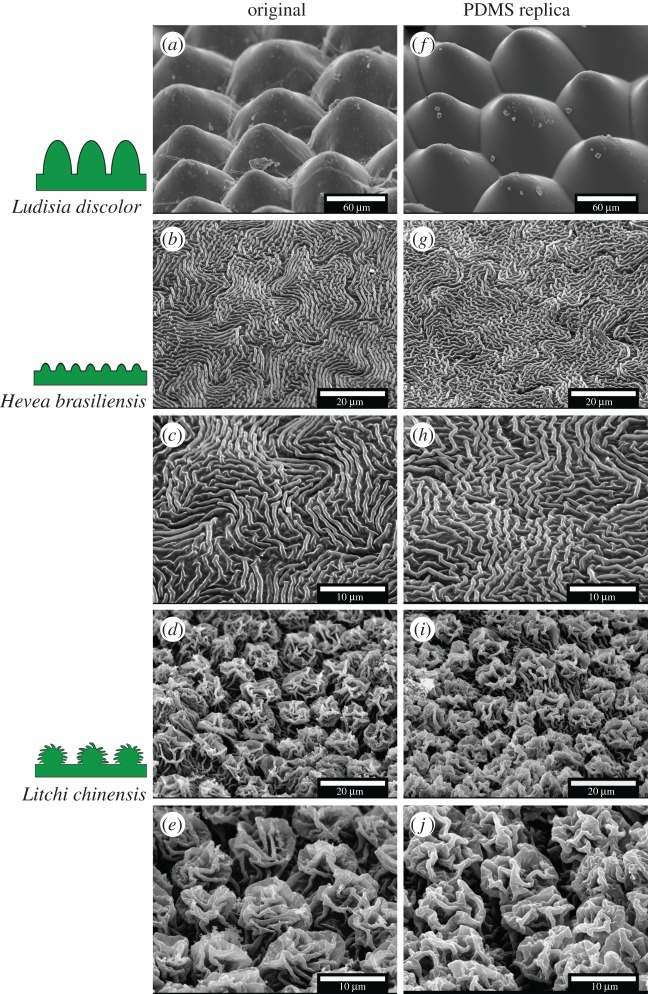
SEM images of original plant leaf surfaces (*a*–*e*) and their PDMS polymeric replicas (*f*–*j*). Pictograms on the left side represent the type of structuring. (*a*,*f*) *Ludisia discolor* (adaxial leaf surface; coarse cone-like surface structuring). (*b*,*g*,*c*,*h*) *Hevea brasiliensis* (adaxial leaf surface; fine fold-like microstructures). (*d*,*i*,*e*,*j*) *Litchi chinensis* (abaxial leaf surface; hierarchical surface structures). (*c*,*h*,*e*,*j*) Represent the higher magnification images of (*b*,*g*) and (*d*,*i*), respectively.

In the next step, epoxy negative moulds (herein called negative moulds) developed from *H. brasiliensis*, *L. discolor,* and from *L. chinensis* leaves (after KOH treatment) were further used to replicate leaf surface patterns onto PDMS surfaces. Two-component PDMS elastomer (Bluesil ESA 7250 A & B kit, Bluestar Silicones GmbH, Leverkusen, Germany) was uniformly mixed (weight ratio of monomer to cross-linker of 10 : 1) in a plastic cup for about 5 min, using a glass rod. Then the mixture was kept in a vacuum desiccator for 30 min and was degassed (2–3 times) to remove trapped air bubbles in the mixture. The clear and bubble-free mixture was slowly poured onto the negative moulds from a corner to limit the formation of bubbles, so that the mixture flows all over the mould surface (figure 1*f*). Negative moulds filled up with PDMS mixture were kept in a vacuum chamber for 1 h to remove air entrapped at the interface between PDMS and micro/nano-cavities of the negative mould (figure 1*g*). The samples were kept in a heating oven at 60°C for 4 h and then the PDMS replicas were gently peeled off from the negative moulds (figure 1*h*). The PDMS replicas were washed with isopropyl alcohol (≥99.95%, Carl Roth GmbH & Co. KG, Karlsruhe, Germany) in an ultrasonicator for 10 min, to wash off any residual particles and followed by drying with a compressed air stream.

### 2.3. Surface characterization

Visualization and characterization of surface morphology of the plant surfaces and their polymer replicas were performed using scanning electron microscopy (SEM). For SEM examination of the leaf surfaces, fresh leaves were dehydrated with methanol and dried by using critical point drier (LPD 030, Bal-Tec) [33]. Prior to SEM investigation, all samples (plant leaves and their replicas) were mounted on aluminium stubs (Plano GmbH, Wetzlar, Germany) using double-sided adhesive conducting tabs (Plano GmbH, Wetzlar, Germany). In addition to this, side walls of polymer replica samples were coated with highly conductive silver paint (Acheson Silver DAG 1415M, Plano GmbH, Wetzlar, Germany) to form an electron conducting path to the stubs. All samples were sputter coated with a thin (15–20 nm) layer of gold (Cressington Sputter Coater, 108 auto). Afterwards, all samples were examined using a Leo 435 vp scanning electron microscope (Leica, Wiesbaden, Germany). All SEM examinations were performed at 45° tilting angle, at an accelerating voltage of 15 kV.

## 3. Results and discussion

SEM images of the surfaces of original plant leaves and of their replicas are presented (side by side for better comparison) in figure 2 and illustrate the high precision of the developed replication process. The surface of the PDMS replica of *L. discolor* (figure 2*f*) shows microstructures (circular cones) very similar in size and shape to the original leaf surface structures (figure 2*a*). No explicit shrinkage or shape damage of the convex microstructures on the replica surface was observed. The surfaces of fresh *L. discolor* leaves show a shiny (glossy) optical appearance and the same optical appearance was also observed on its replica surface. The result of replications of the *H. brasiliensis* leaf surface shows that very fine (individual folds with a height and width of less than 1 µm) surface structures were successfully replicated without any fusion or overlapping of individual folds, as proved in figure 2*b* and figure 2*g*. High-resolution images of the *H. brasiliensis* leaf surface and its replica are shown in figure 2*c* and figure 2*h* confirming the high precision and spatial resolution of the new replication process. In the present investigation, the most remarkable replication result was obtained for *L. chinensis,* where complex hierarchical structures with undercuts and overhanging sub-structures could be replicated precisely (figure 2*d* and figure 2*i*). It is interesting to see in high-resolution images (figure 2*e* and figure 2*j*) that ‘rose flower-shaped' patterns on the *L. chinensis* leaf surface were transferred to the replica undamaged. Such type of complex structures with undercuts is usually difficult to replicate without breaking the overhanging folds while peeling the replica from the negative mould.

This qualitative comparison based on SEM surface images provides evidence for the high precision and versatility of the replication technique, which is achieved due to the high compliance of PDMS (elastic modulus, *E* ≃ 2 MPa [44,48]) in comparison with the elastic rigid behaviour of the cured epoxy resin. Moreover, the very low viscosity (≃400 mPa s) of uncured epoxy resin benefits to the better filling of liquid epoxy into the fine and complex leaf structures. This technique was even successful in the case of *L. chinensis* leaves as the epoxy mixture could completely and utterly fill the fine undercut cavities on the leaf surface. However, after complete curing of the moulding material, complex structures of leaf were tangled up in the moulding mass, with the consequence that the leaf surface was un-separably embedded (inlaid) in the cured epoxy. Therefore, the strong alkaline chemical treatment as described in detail in replication procedure section was successfully used to dissolve the plant material at the interface between leaf and epoxy mould. The low viscosity of liquid PDMS mixture also helps when filling up the negative moulding material (epoxy) (figure 1*f*). After polymerization (i.e. curing), the PDMS becomes a soft rubber type material. The present results strongly point towards the interpretation that the pronounced difference in the elasticity of both materials (epoxy and PDMS) is essential for easy removal of the positive replica by a simple peeling process. As a consequence, the flexible nature of PDMS prevents breaking of overhanging and damaging undercut structures. Prüm *et al.* [5] performed similar replications of complex microstructured *L. chinensis* leaf surface by using a PVS-Epoxy approach. However, some structural imperfection can be observed particularly on the overhanging cuticular folds of ‘rose flower-shaped’ hierarchical patterns [5]. This might be due to the quick curing and high viscosity of PVS impression material, which results in an incomplete filling of PVS into the undercuts cavities of the complex structure of *L. chinensis.* Our replication approach overcomes this limitation, thanks to the low viscosity of both epoxy and PDMS mixture prior to polymerization. Moreover, in some of the previously published direct replication approaches [25,26,29,30], plant leaves are exposed to vacuum or high temperatures which can produce replication errors due to shrinkage or collapse of surface structures. In the present work, no plant material was exposed to vacuum preventing early shrinkage. Furthermore, all the epoxy moulds were developed at room temperature (as previously described) and because the plant samples were completely covered with epoxy resin, water loss by means of evaporation is minimized. In addition, the temperature of the moulding mass (epoxy resin) was continuously recorded during the curing process with a thermocouple probe. No noteworthy variation in ambient temperature was observed (24 ± 2°C).

**Figure 3.**
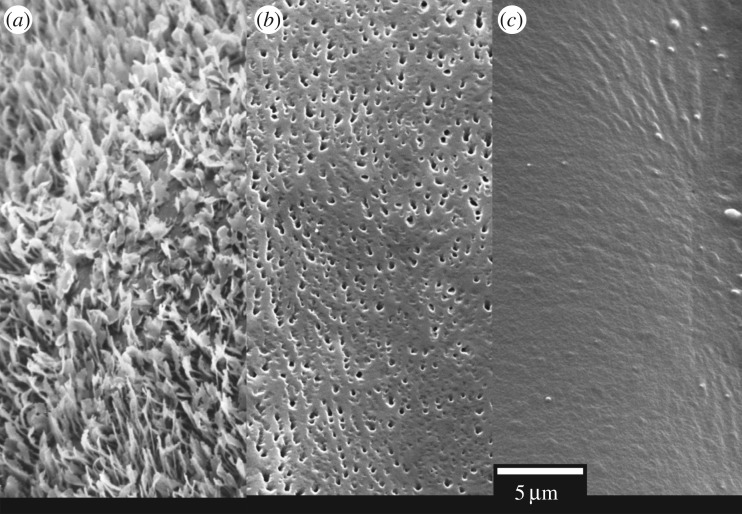
High-resolution SEM image of original leaf surfaces (*a*) of *Iris germanica* (adaxial leaf surface; covered with a dense network of perpendicularly oriented wax platelets), developed negative epoxy mould (*b*) and their PDMS polymeric replica (*c*).

Up to this point, the versatile replication abilities of our technique have been demonstrated. However, this technique shows some limitations which have to be discussed. The replication of a fourth plant leaf was studied to illustrate these issues. Actually, *Iris germanica* (bearded iris) plant leaf (adaxial, i.e. upper leaf surface) was chosen due to its particular surface structure made of a three-dimensional dense arrangement of perpendicularly oriented long wax platelets. One may note that the freshly hydrated leaf was used for SEM observation to avoid any wax platelets destruction that might be caused by methanol dehydration protocol. The sample was quickly examined before any desiccation artefacts were observed [49]. Figure 3*a* shows the high-resolution image of the native *I. germanica* leaf surface covered with the dense network of high aspect ratio wax platelets, and its PDMS replica is shown in figure 3*c*. It shows clearly the replication flaw for the wax platelets morphology. To investigate the cause of this replication limitation, the SEM image of the negative epoxy mould is reported in figure 3*b*.

As can be seen on negative epoxy mould, some wax morphology partially formed a negative imprint. We believe that one possible reason might be that the liquid epoxy mixture stays on top of some wax platelets and could not completely penetrate inside the wax structures as no external pressure was applied on the moulding mass. Consequently, to this partial wetting of the fresh leaf surface by the epoxy mixture, only some wax patterns were correctly transferred onto the negative mould. We also speculate that the fragile and high-aspect-ratio wax platelets might have embedded within the cured epoxy mould and broken down during separation. It is worthy of anticipation that some leaves surface with high aspect ratio complex structures or with long, pointy slender members-like structures (such as the long hairy with pointy branches trichome microstructures on *Arabidopsis thaliana* leaf) might be difficult to replicate [50], in particular while separating of the trichomes from negative moulds.

Finally, although this new replication technique has some limitations, it demonstrates good replication abilities for various surfaces and offers extra advantageous aspects of the Epoxy–PDMS usage: (1) The negative epoxy moulds do not require any intermediate anti-stiction surface treatment (e.g. deposition of self-assembled silane monolayers), which is compulsory in some other processes such as PDMS–PDMS replication [35,38,51]. These additional surface treatments are usually time-consuming, need extra equipment and could give rise to complications. Moreover, the present replication approach eliminates the risk of chemical contamination on the positive PDMS replicas, because no chemical surface treatments have to be performed on the negative epoxy mould. (2) Our replication approach does not immensely depend on the process parameters and appears highly stable against variation in ambient conditions. This replication process can be easily performed without using costly and sophisticated laboratory machines. (3) Cured negative epoxy moulds which are hard and robust after curing (Young's modulus of the moulds approx. 3 GPa) offer long durability and high stability even of delicate surface microstructures. The same epoxy mould without any further treatment can be used repeatedly to fabricate multiple PDMS replicas.

## 4. Conclusion

The newly developed replication approach presented here provides a simple, inexpensive, durable and precise way to directly transfer coarse, fine (with a lateral resolution down to sub-micron), as well as complex hierarchical geometries from biological surfaces onto PDMS soft polymer. This technique can be used for the rapid development of bioinspired functional surfaces and can also be upscaled on a large area (cm^2^), although limited to the size of leaves. Replicas developed by this technique have a major perspective to investigate the role of topography on the surface functionalities such as optical properties, wetting properties, tribological properties, antifouling properties, etc. Therefore, the technique presented in this work represents a relevant alternative for the micro-replication of biological surface structures.

## Data accessibility

All data required to attempt a replication are provided within the manuscript, specifically in the Material and methods section and the figures presented.
